# PHB3 Is Required for the Assembly and Activity of Mitochondrial ATP Synthase in Arabidopsis

**DOI:** 10.3390/ijms24108787

**Published:** 2023-05-15

**Authors:** Qingqing Wei, Baoyin Chen, Junjun Wang, Manna Huang, Yuanye Gui, Aqib Sayyed, Bao-Cai Tan

**Affiliations:** Key Laboratory of Plant Development and Environment Adaptation Biology, Ministry of Education, School of Life Sciences, Shandong University, Qingdao 266237, China; weiqq93@163.com (Q.W.); ba_oyin_love@126.com (B.C.); 201912282@mail.sdu.edu.cn (J.W.); huangmanna1990@126.com (M.H.); guiyuanye2019@163.com (Y.G.); aqib.sayyed@yahoo.com (A.S.)

**Keywords:** Arabidopsis, mitochondria ATP synthase, assembly factor, PHB3

## Abstract

Mitochondrial ATP synthase is a multiprotein complex, which consists of a matrix-localized F1 domain (F1-ATPase) and an inner membrane-embedded Fo domain (Fo-ATPase). The assembly process of mitochondrial ATP synthase is complex and requires the function of many assembly factors. Although extensive studies on mitochondrial ATP synthase assembly have been conducted on yeast, much less study has been performed on plants. Here, we revealed the function of Arabidopsis prohibitin 3 (PHB3) in mitochondrial ATP synthase assembly by characterizing the *phb3* mutant. The blue native PAGE (BN-PAGE) and in-gel activity staining assays showed that the activities of ATP synthase and F1-ATPase were significantly decreased in the *phb3* mutant. The absence of PHB3 resulted in the accumulation of the Fo-ATPase and F1-ATPase intermediates, whereas the abundance of the Fo-ATPase subunit a was decreased in the ATP synthase monomer. Furthermore, we showed that PHB3 could interact with the F1-ATPase subunits β and δ in the yeast two-hybrid system (Y2H) and luciferase complementation imaging (LCI) assay and with Fo-ATPase subunit c in the LCI assay. These results indicate that PHB3 acts as an assembly factor required for the assembly and activity of mitochondrial ATP synthase.

## 1. Introduction

Mitochondria produce the bulk of the energy used by almost all eukaryotic cells through oxidative phosphorylation (OXPHOS), which is composed of five membrane complexes (Complex I–V) [1,2]. Complex I–IV, defined as the electron transport chain [3,4,5], can pump protons from the mitochondrial matrix to the intermembrane space (IMS) and undertake the task of producing a proton concentration gradient [6,7]. After that, Complex V (also named ATP synthase or ATPase) harvests the electrochemical energy from the proton motive force across the mitochondrial inner membrane and catalyzes the synthesis of ATP from ADP and phosphoric acid by proton translocation and subunit rotation [8,9,10,11,12]. Therefore, mitochondrial ATP synthase plays a key role in energy production.

Mitochondrial ATP synthase is a multiprotein complex, which is mainly divided into two domains, a matrix-soluble F1 domain (F1-ATPase) and an inner membrane-embedded Fo domain (Fo-ATPase). In yeast, the F1 domain consists of five kinds of subunits: α, β, γ, δ, and ε. The heterohexamer (αβ)_3,_ composed of subunits α and β, forms the catalytic head, and the subunits γ, δ, and ε constitute the central stalk [13]. The Fo domain includes subunits a, b, c, d, e, f, g, h, i/j, k, 8, and OSCP. The subunit c oligomerizes to form a ring-like homologous polymer, named c-ring, which attaches to subunit a to form a stator [14]. The rest of the Fo domain subunits form a peripheral stalk to fasten the F1 domain into the Fo domain stator. The peripheral stalk and central stalk are considered to be parts of the Fo domain and F1 domain, respectively [15,16,17]. The structure and composition of mitochondrial ATP synthase are highly conserved among species and have been well-studied in yeast and animals [18,19]. However, the assembly mechanism of mitochondrial ATP synthase remains unclear, especially in plants.

The precise assembly process of mitochondrial ATP synthase is extremely complicated and difficult to study. First, this process involves multiple subunits and intermediates, which are rapidly updated [20,21,22,23,24]. In this case, it is difficult to detect the assembly intermediates, especially the high-turnover ones. Second, mitochondrial ATP synthase subunits are encoded by both the mitochondrial and nuclear genomes. Therefore, the assembly process involves the expression, processing, and translocation of subunits from these two sources [25,26,27]. In humans, the partial assembly process of mitochondrial ATP synthase is raised. Briefly, the F1 domain can insert into the c-ring to form the F1-c-ring intermediate, and the free F1 domain and F1-c-ring intermediate can both combine with the peripheral stalk. Finally, the subunits e-f-g intermediate helps the subunits a and 8 to insert into the space between the c-ring and peripheral stalk [21,22]. The assembly of mitochondrial ATP synthase in yeast is partially different from humans. In *Saccharomyces cerevisiae*, the F1 domain firstly combines with the assembly intermediate that contains the peripheral stalk, subunits a and 8. The c-ring thus associates with subunit a to allow the formation of the ATP synthase [20,27]. In contrast, there are few studies on mitochondrial ATP synthase assembly in plants. In Arabidopsis, Röhricht et al. found that the F1 domain is assembled independently by complexome profiling [28]. For the Fo domain, subunits 8 and i/j are first assembled, followed by subunit b. Then, they connect other subunits of the Fo domain except for OSCP. This intermediate and F1 domain form ATP synthase, which is finally assembled by subunit OSCP [28]. However, the assembly process of Arabidopsis mitochondrial ATP synthase remains uncertain, lacking accurate experimental evidence.

Previous studies reported that multiple assembly factors participate in the assembly process of yeast mitochondrial ATP synthase [29,30,31,32,33,34]. For the F1 domain, assembly factors ATP11 and ATP12 proven that they can bind to subunits β and α, respectively, to form the (αβ)_3_ hexamer [30,31,32]. The assembly of the Fo domain is more complicated compared with the F1 domain due to more subunits and intermediates. In this case, more assembly factors are required. In yeast, ATP25 can promote the expression of subunit c and the formation of the c-ring oligomer [35]. For subunit a, two molecular chaperones are involved in its processing and assembly, which are ATP23 and ATP10. ATP23 is a metalloprotease, which is responsible for the cleavage of the first 10 amino acids at the N-terminal of newly synthesized subunit a. The loss of ATP23 results in the degradation of the subunit a precursor, and blocks the assembly of the Fo domain. ATP10 mainly assists ATP23 in processing the subunit a precursor [36,37,38,39]. Furthermore, INAC (inner membrane assembly complex) is found to promote the combination of subunit a and the c-ring [20,27]. OXA1 can directly interact with newly synthesized subunit c and is also required to maintain the assembly competence of the F1-c-ring subcomplex for its association with subunit a [40]. However, the deletion mutations of these ATP synthase assembly factors might be lethal, such as the mutants *atp11* and *atp12* in Arabidopsis [41]. So far, no exact functions of assembly factors have been reported in plants. Therefore, the identification of assembly factors of mitochondrial ATP synthase in plants is necessary.

Prohibitins (PHBs) are originally discovered as tumor-suppressor genes in mammalian cells [42] and are widely distributed in cells, including mitochondria, nucleus, and plasma membrane [42,43]. In mammals and yeast, there are two members of PHB proteins, PHB1 and PHB2, which form a ring-shaped complex on the inner mitochondrial membrane. The complex functions as a universal protein scaffold for mitochondrial protein processing and respiratory chain function [44,45]. In yeast, the PHB proteins are found to genetically interact with assembly factors ATP23 and ATP10 to regulate the degradation of subunit a precursor [37]. In Arabidopsis, there are seven *PHB* genes, *PHB1*–*PHB7*, among which *PHB5* and *PHB7* are pseudogenes. Proteins encoded by the rest genes are divided into type I (PHB3 and PHB4) and type II (PHB1, PHB2, and PHB6) classes, corresponding to yeast PHB1 and PHB2, respectively [46,47]. PHB3 is localized in several cellular locations, including the nucleus and mitochondria, and the loss of the function of PHB3 results in a slow-growth phenotype in Arabidopsis [47,48,49]. Previous studies indicate that PHB3 is involved in cell production, cell proliferation, phytohormone signal transduction, and so on [50,51,52,53]. Transmission electron microscopy (TEM) results reveal that the mitochondria are swollen, and the inner mitochondrial membrane cristae disappear in the *phb3* mutant [47]. In the absence of PHB3, alternative oxidase genes (*AOX1A* and *AOX1C*) and NAD(P)H dehydrogenase genes (*NDA1*, *NDB2*, *NDB3,* and *NDB4*) of alternative pathways are induced [51]. In plants, the induction of alternative pathway genes is considered as the retrograde signals when mitochondria are impaired, implying that the mitochondria are damaged in the *phb3* mutant.

In this paper, we elucidated the mitochondrial function of PHB3 in Arabidopsis. We found that the deletion of PHB3 resulted in the significantly reduced activities of mitochondrial ATP synthase and F1-ATPase. Western blotting showed that the abundance of subunit a was increased in Fo-ATPase, while decreased in the ATP synthase monomer in the *phb3* mutation. In addition, the loss of PHB3 leads to the accumulation of F1-ATPase by hybridization with primary antibodies against subunits α and β. Meanwhile, we performed Y2H and LCI assays and discovered that PHB3 could interact with the subunit c of Fo-ATPase, and the subunits β and δ of F1-ATPase. Together, our studies provided insight into the assembly of mitochondrial ATP synthase in Arabidopsis. PHB3 might act as an assembly factor and is required for the assembly and activity of mitochondrial ATP synthase.

## 2. Results

### 2.1. Loss of Function of PHB3 Impairs the Abundance of Multiple Mitochondrial Proteins

**Figure 1:**
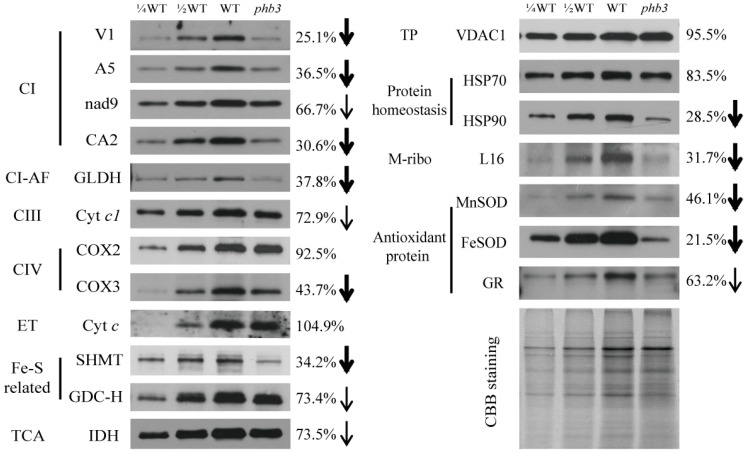
The abundance of mitochondrial proteins in the *phb3* mutant. Crude mitochondrial total proteins of the wild-type and the *phb3* mutant were separated by SDS-PAGE and transferred to the polyvinylidene difluoride (PVDF) membrane. Western blotting analysis of total mitochondrial proteins with antibodies against various mitochondrial proteins. CI, complex I; CI-AF, complex I assembly factor; GLDH, l-galactone-1,4-lactone dehydrogenase; CIII, complex III; CIV, complex IV; ET, electron transport; Cyt *c*, cytochrome *c*; GDC-H, glycine decarboxylase-H protein; SHMT, serine hydroxymethyltransferase; TCA, tricarboxylic acid cycle; IDH, isocitrate dehydrogenase; HSP, heat shock protein; TP, transport pathway; VDAC1, voltage-dependent anion-selective channel protein 1; M-ribo, mitochondria ribosome protein; MnSOD, Mn superoxide dismutase; FeSOD, Fe superoxide dismutase; GR, glutathione reductase. The intensity value of the immune signals in the wild-type and the *phb3* mutant are measured by ImageJ software (version 1.46r). The percentage of relative intensity value behind each group indicates the relative abundance of the *phb3* mutant to the wild-type (*phb3*/WT). Thick arrows represent a more than 2-fold decrease in the abundance in the *phb3* mutant, and thin arrows represent a 1.5–2 fold decrease. CBB (coomassie brilliant blue) staining gels were used as the sample loading control.

To further explore the biological function of Arabidopsis *PHB3* (*AT5G40770*) in mitochondria, we obtained the T-DNA mutant, *phb3* (SALK_020707), harboring an insertion in the first exon of this gene [51,52]. We first analyzed the abundance of several mitochondrial proteins in the *phb3* mutants. These proteins are involved in various metabolic pathways in mitochondria, such as the complex assembly, electron transport, TCA cycle, and antioxidant system. Total mitochondrial proteins of the wild-type and *phb3* mutants were extracted, separated in SDS-PAGE, and analyzed with the Western blotting assay. As shown in Figure 1, the levels of most proteins were decreased in the *phb3* mutant compared with the wild-type (Figure 1). Among these, the subunits of mitochondrial complex I (V1, A5, and CA2) [54,55], complex IV (COX3), as well as complex I assembly factor GLDH (l-galactone-1,4-lactone dehydrogenase) [54,55] in abundance were significantly reduced (Figure 1). The levels of SHMT (serine hydroxymethyltransferase) [56], heat shock protein HSP90 [57], and mitochondrial ribosomal protein L16 were also significantly decreased, together with the potent antioxidant MnSOD and FeSOD [58] (Figure 1). In contrast, several proteins were marginally decreased in the *phb3* mutant (Figure 1), including the complex I subunit Nad9 [55], complex III subunit Cyt *c*1 [59], GDC-H (mitochondrial glycine decarboxylase complex) [60], IDH (isocitrate dehydrogenase) [61], and GR (glutathione reductase) [62]. This result indicates that mitochondrial function is impaired in the *phb3* mutant.

### 2.2. The Loss of PHB3 Results in the Decrease in Mitochondrial ATP Synthase Activity

**Figure 2:**
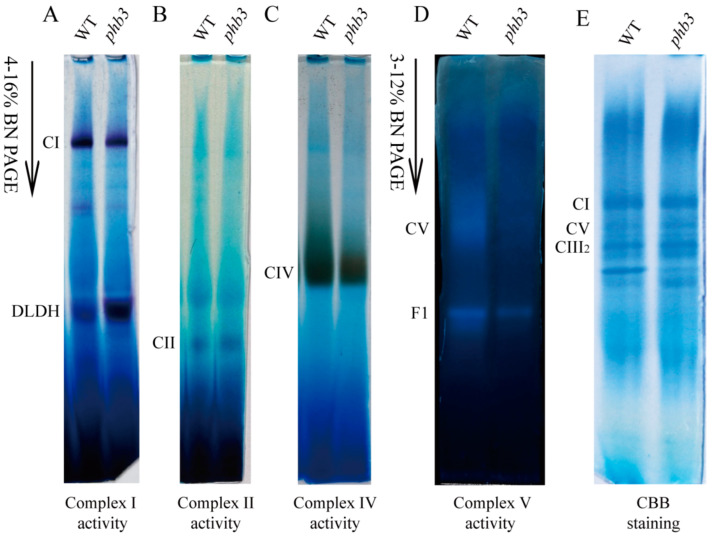
The loss of function of PHB3 affects the activity of mitochondrial ATP synthase. Mitochondrial proteins were isolated from 12 day-old seedlings of wild-type (WT) and *phb3* mutant grown in the dark. The proteins were solubilized with n-Dodecyl β-D-maltoside (β-DM) followed by BN-PAGE. (**A**–**D**) The activities of respiratory chain complexes were visualized by in-gel activity staining. (**E**) CBB staining after electrophoresis shows the equal loading of protein. The bands of complex I (CI), dihydrolipoamide dehydrogenase (DLDH), complex II (CII), complex IV (CIV), complex V/ATPase (CV), and F1-ATPase (F1), complex III dimer (CIII_2_) are indicated.

The main function of mitochondria is to generate energy via the mitochondrial respiratory chain [2,3]. Therefore, we investigated whether the loss of Arabidopsis PHB3 affects the function of the mitochondrial respiratory complex. Mitochondria were isolated from the wild-type and *phb3* mutant seedlings grown in the dark for 12 days, as previously described [63]. The mitochondrial membrane complexes were solubilized with n-Dodecyl β-D-maltoside (β-DM) and then separated by blue native polyacrylamide gel electrophoresis (BN-PAGE), and the in-gel complex activity was analyzed [64]. Coomassie brilliant blue (CBB) staining showed an equal protein loading (Figure 2E). The results showed that the activity of ATP synthase was decreased in the *phb3* mutant compared with the wild-type (Figure 2D). The F1-ATPase activity was also reduced (Figure 2D), while the activities of complex I and complex IV were slightly decreased (Figure 2A,C). In addition, no significant differences in the activity of complex II between the *phb3* mutant and wild-type were observed (Figure 2B). These results indicate that the loss of function of *PHB3* affects mitochondrial ATP synthase.

### 2.3. PHB3 Is Essential for the Assembly of Mitochondrial ATP Synthase

**Figure 3:**
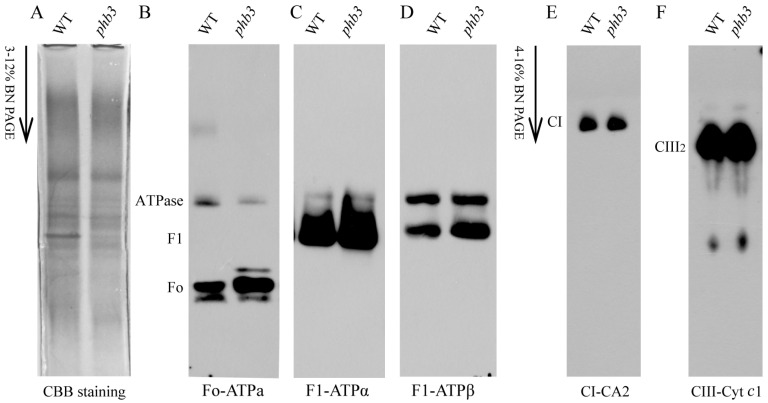
The loss of PHB3 influences the assembly of ATP synthase. Mitochondrial proteins of 12 day-old wild-type (WT) and the *phb3* mutant were solubilized with β-DM and then separated by BN-PAGE. (**A**) CBB staining and immunoblot analysis with the following primary antibodies: (**B**) Fo-ATPa, subunit a of Fo-ATPase; (**C**) F1-ATPα, subunit α of F1-ATPase; (**D**) F1-ATPβ, subunit β of F1-ATPase; (**E**) CI-CA2, subunit CA2 of complex I; (**F**) CIII-Cyt *c*1, subunit cyt *c*1 of complex III. The positions of complex I, III, and V and the corresponding intermediates are indicated. Fo, Fo domain of ATP synthase. F1, F1 domain of ATP synthase.

The reduced activity of the mitochondrial ATP synthase in *phb3* mutants might be caused by defects in its assembly. To test this possibility, we analyzed the abundance of the ATP synthase subunits in the blue native gels using Western blotting (WB). CBB staining gels were used as the sample loading control (Figure 3A). Antibodies against the Fo-ATPase subunit a (Fo-ATPa), F1-ATPase subunit α (F1-ATPα), and subunit β (F1-ATPβ) were used. The result showed that the abundance of Fo-ATPase subunit a in ATP synthase monomer was decreased in the *phb3* mutant compared with the wild-type, whereas the level of Fo-ATPase was increased (Figure 3B). This result suggests that subunit a of Fo-ATPase cannot be efficiently assembled into the intact ATP synthase in the absence of PHB3. In contrast, the abundance of F1-ATPα and F1-ATPβ in ATP synthase did not change in the *phb3* mutant, while both were significantly increased in F1-ATPase (Figure 3C,D). These results indicate that the assembly process of ATP synthase is impaired in the *phb3* mutant, probably due to the blocked assembly of the Fo-ATPase into ATP synthase. On the other hand, the PHB3 deficiency resulted in the decreased abundance of Fo-ATPa in ATP synthase (Figure 3B) but not the abundance of F1-ATPα and F1-ATPβ (Figure 3C,D), implying that the ATP synthase lacks subunit a in the *phb3* mutant. Meanwhile, we detected the presence of complex I subunit CA2 and complex III_2_ subunit Cyt *c*1, and their distribution remained unchanged between the *phb3* mutant and wild-type (Figure 3E,F). Together, these results suggest that PHB3 is involved in the assembly of ATP synthase.

### 2.4. PHB3 Interacts with ATP Synthase Subunits

**Figure 4:**
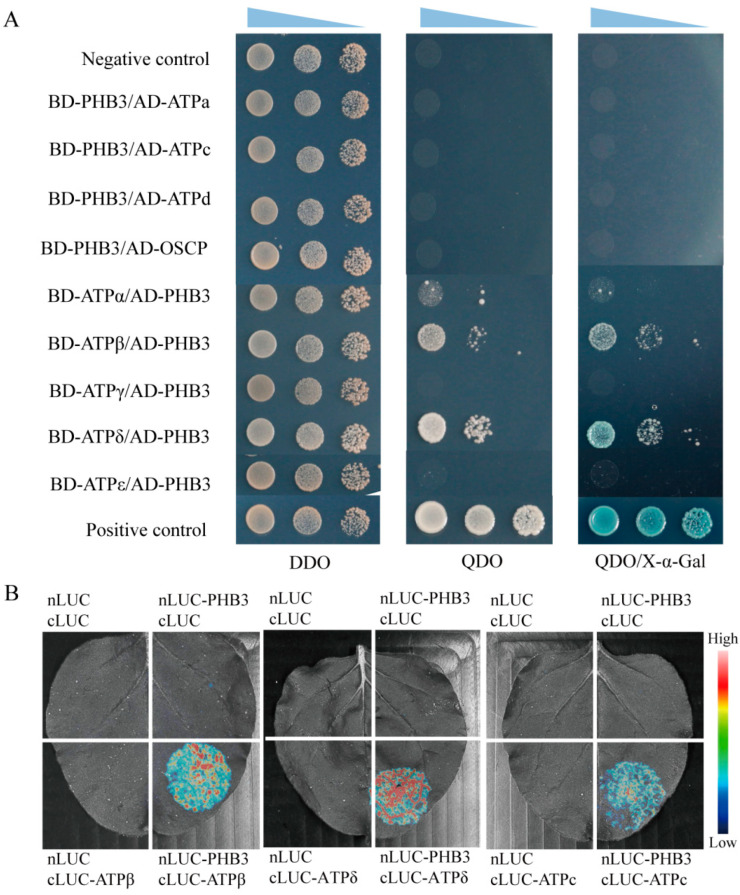
Interactions between PHB3 and ATP synthase subunits by Y2H system and LCI assay. (**A**) Y2H analysis of the interactions between PHB3 and Fo-ATPase subunits (ATPa, ATPc, ATPd, OSCP) or F1-ATPase subunits (ATPα, ATPβ, ATPγ, ATPδ, and ATPε). The yeast cells of strain Y2H Gold harboring the indicated plasmid combinations grown in DDO (SD-Trp/-Leu), QDO (SD-Trp/-Leu/-His/-Ade), and QDO/X-α-Gal (QDO with X-α-Gal filter) media were indicated. Cotransformation of pGBKT7-T with pGBKT7-53 was used as a positive control. A pair of plasmid combinations of empty pGBKT7/pGADT7 were used as a negative control. (**B**) Interaction analysis by LCI assay in *Nicotiana benthamiana* leaf epidermal cells. PHB3 protein was fused to the N-terminal fragment of firefly LUC (nLUC). Subunits ATPβ, ATPδ, and ATPc were fused to the c-terminal fragment of firefly LUC (cLUC). The results were from three separate biological replications.

In yeast, mitochondrial ATP synthase assembly factors ATP11 and ATP12 can bind to the subunits β and α, respectively [29,30], promoting the formation of (αβ)_3_ hexamer in F1-ATPase [32]. In Arabidopsis, the homologs of ATP11 and ATP12 also interact with the corresponding subunits [41]. Our results show that the loss of the PHB3 function impairs the assembly and activity of the mitochondrial ATP synthase (Figure 2D and Figure 3B). Therefore, it is reasonable to speculate that PHB3 might interact with some mitochondrial ATP synthase subunits. To test this notion, we first examined the interaction between PHB3 and ATP synthase subunits using the yeast two-hybrid (Y2H) system. The open reading frame (ORF) sequences of Fo-ATPase subunits (ATPa, ATPc, ATPd, OSCP) and PHB3 were fused to the expression vector pGADT7 (AD). The ORF sequences of F1-ATPase subunits (ATPα, ATPβ, ATPγ, ATPδ, and ATPε) and PHB3 were cloned into the expression vector pGBKT7 (BD). The results showed that PHB3 interacted with subunits ATPβ and ATPδ of F1-ATPase in the Y2H system (Figure 4A). We also performed the luciferase complementation imaging (LCI) assay in the *Nicotiana benthamiana* leaf epidermal cells. The result showed that the co-expression of nLUC-PHB3/cLUC-ATPβ and nLUC-PHB3/cLUC-ATPδ in tobacco leaves reconstituted strong luciferase activities, compared with the negative controls (Figure 4B). These results indicate that PHB3 could interact with the F1-ATPase subunits β and δ in Arabidopsis.

In the Y2H system, PHB3 did not interact with subunit a (Figure 4A). However, the absence of PHB3 seriously affected the assembly of Fo-ATPase subunit a, resulting in its accumulation in Fo-ATPase and reduction in ATP synthase (Figure 3B). Therefore, it is possible that PHB3 affects the assembly of subunit a through its neighboring subunits, i.e., other Fo-ATPase subunits. Subunit a and the c-ring are attached in the mitochondrial inner membrane and form a proton translocating channel at their binding interface [65]. Therefore, we further tested the interaction between PHB3 and subunit c. The results showed that PHB3 could interact with subunit c in the LCI assay (Figure 4B). These results imply that PHB3 directly binds to subunit c, which is required for the assembly between subunit a and the c-ring. When PHB3 is absent, subunit a either cannot bind to the c-ring or the binding is unstable. Consequently, it leads to the decreased abundance of subunit a in the ATP synthase monomer (Figure 3B). In conclusion, PHB3 affects the assembly process of mitochondrial ATP synthase, possibly through direct binding to its subunits.

## 3. Discussion

### 3.1. PHB3 Acts as an Assembly Factor in the Assembly of Mitochondrial ATP Synthase

**Figure 5:**
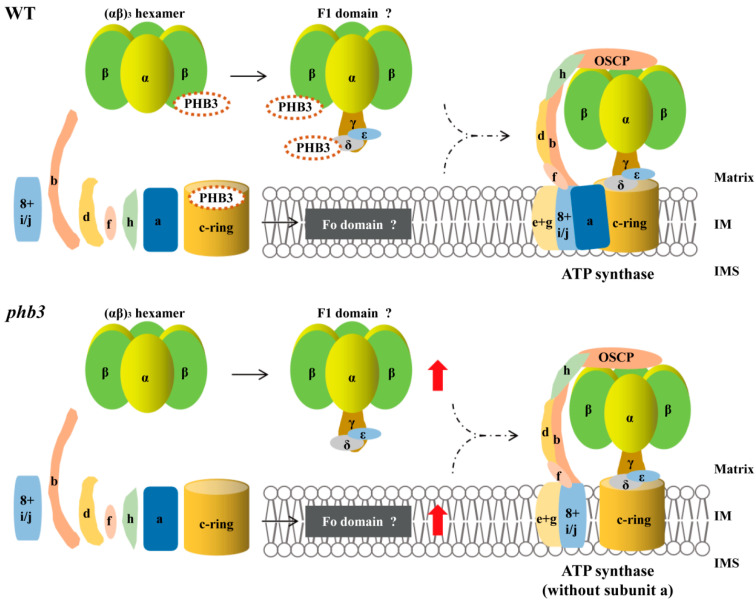
Postulated model of PHB3 in the assembly of Arabidopsis mitochondrial ATP synthase. This model refers to Röhricht et al. [28]. Subunits are labeled with yeast nomenclature. Mitochondrial ATP synthase consists of the cytoplasmic F1 domain and membrane-embedded Fo domain. The top panel shows the assembly of mitochondrial ATP synthase in the presence of PHB3 and its potential further interaction with the subunits Fo-ATPc, F1-ATPβ, and F1-ATPδ. The subunits α and β consititute (αβ)_3_ hexamer. The central stalk (subunits γ, δ, and ε) and the (αβ)_3_ hexamer together form the F1 domain located in the mitochondrial matrix. Fo domain is composed of subunits 8, i/j, b, d, f, h/F_A_d, a, and the c-ring. The Fo domain and the F1 domain connect to form the ATP synthase monomer in the bottom panel where there is no PHB3. F1 domain and Fo domain are accumulated. The structure of ATP synthase monomer lacks subunit a. The red arrows represent the increase in abundance. The dotted lines indicate that some assembly steps are omitted. Matrix, mitochondrial matrix. The question marks after F1 domain and Fo domain indicate that these two structures are probable. IM, inner mitochondrial membrane; IMS, intermembrane space.

PHB3 has been localized in the mitochondrion and nucleus [47,48]. Nuclear-localized PHB3 acts as a negative or positive co-regulator of transcription, affecting cell cycle and cell proliferation to regulate plant development [50,51,52,53]. However, its mitochondrial function remains unclear. In this study, we found that PHB3 may function as an assembly factor of the mitochondrial ATP synthase and is required for the assembly of subunit a from the Fo domain to ATP synthase (Figure 5). Two pieces of evidence support this conclusion. First, our results showed that the loss of PHB3 blocked the assembly of the Fo domain into ATP synthase via subunit a (Figure 3B) by interacting with its neighboring subunit c (Figure 4B). In yeast, OXA1 is an assembly factor of mitochondrial ATP synthase, which connects with subunit c. In the *oxa1* mutant, the c-ring combines with the F1 domain to form the F1-c-ring subcomplex, while a further assembly of the F1-c-ring with subunit a is limited [40]. This is consistent with the molecular phenotype of PHB3 deficiency in this study (Figure 5). In addition, the function of the assembly factor of mitochondrial ATP synthase in yeast, INA22 (inner membrane assembly protein 22) [20], lends support to this conclusion as well. In yeast, INA22 is required for the combination of the c-ring and subunits a/8 by directly binding to these subunits. Mutation of *INA22* leads to an accumulation of the assembly intermediate containing subunits a/8. Meanwhile, the subunit a is decreased in the monomer of ATP synthase [20]. Similarly, these changes were also found in the *phb3* mutant using the immunoblotting assay with anti-ATPa (Figure 3B). In addition, ATP23 and ATP10 mediate the assembly of subunit a into the Fo domain in the assembly of ATP synthase. The loss of ATP23 and ATP10 promotes the accumulation of the F1 domain and Fo-containing assembly intermediates but decreases the abundance of ATP synthase [36,37,38], which is analogous to the results observed in the *phb3* mutant (Figure 3B–D). Second, PHB3 is not present in the assembled ATP synthase, i.e., not a subunit of the mature holoenzyme. In Arabidopsis, PHB3 mainly forms a ~1 MDa complex with other PHB proteins (PHB1, PHB2, PHB4, and PHB6), as shown in the mitochondrial complexome data [66]. PHB3 and PHB4 are also detected in the ~1 MDa PHB complex by Western blotting using antibody PHB3/PHB4 [67], showing that the size of the PHB complex is larger than ~620 kDa ATP synthase in Arabidopsis [66]. In that case, a small amount of PHB3 could comigrate with the partial ATP synthase subunits at ~500 and ~100 kDa positions corresponding to the bands in the blue native gels [66]. However, whether PHB3 is present in the assembly intermediates of ATP synthase requires further investigation.

### 3.2. The Loss of PHB3 Decreases the Activity of ATP Synthase and the F1 Domain

Several studies have shown that the loss of F1 domain assembly factors results in the decreased abundance of F1 domain in yeast, such as FMC1 [68,69]. On the contrary, we found that the loss of PHB3 results in the accumulation of the F1 domain and does not affect the assembly of the F1 domain into ATP synthase in Arabidopsis (Figure 3C,D). Furthermore, we also found that the activity of the F1 domain is significantly reduced in the *phb3* mutant (Figure 2D). These results imply that PHB3 is mainly responsible for the activity of the F1 domain rather than its assembly. The phenomenon is probably due to the inactive conformation of the F1 domain subunits β and/or δ without the combination with PHB3 in the *phb3* mutant. Correct folding and modification of F1 domain subunits α and β are essential for the activity of αβ heterodimer in *Acetobacterium woodii* [70]. 

Meanwhile, the activity of ATP synthase was almost undetectable in the *phb3* mutant (Figure 2D), indicating that the decreased activity of ATP synthase may be caused by the decreased activity of the F1 domain and equally by the missing subunit a in the ATP synthase monomer (Figure 3B and Figure 5). In maize, unedited C residue (C635) on the *atp6* gene (encoding subunit a) transcript leads to an amino acid substitution and affects the assembly and activity of ATP synthase [71]. Similarly, the maize chimeric gene *atp6c*, encoding an abnormal ATPa protein with the disordered N-terminal arrangement, results in the decreased activity and abnormal assembly of ATP synthase [72]. These pieces of evidence suggest that subunit a is important for the ATP synthase activity. The severe reduction of ATP synthase activity in the *phb3* mutant resulted from the deficiency of subunit a (Figure 5). In conclusion, we prove that PHB3 is not only involved in the assembly of the Fo domain to ATP synthase, but is also required for the activities of ATP synthase and the F1 domain in Arabidopsis. 

## 4. Materials and Methods

### 4.1. Plant Materials and Growth Conditions

The Arabidopsis *phb3* mutant (SALK_020707) used in this study was in the Col-0 background. Plants were grown either in soil or on half-strength Murashige and Skoog (MS) medium supplemented with 1% sucrose under a 16 h light/8 h dark photoperiod at 22 °C in growth chambers. Tobacco (*Nicotiana benthamiana*) plants were grown in soil with 16 h light at 24 °C.

### 4.2. Total RNA Extraction and cDNA Synthesis

Total RNA was extracted from wild-type seedlings according to the manufacturer’s protocol of the RNeasy Plant Mini Kit (Vazyme Biotech, Nanjing, China). RNA was further digested by RNase-free DNase I (New England Biolabs, Rowley, MA, USA) to remove residual DNA contamination. The reverse transcription was used by the Transcript First-Strand cDNA Synthesis SuperMix (TransGen Biotech, Beijing, China).

### 4.3. Isolation of Mitochondria

Crude mitochondria of 12 day-old Arabidopsis seedlings that were grown on half-strength MS medium in the dark were extracted as described previously [63]. Fresh seedling samples were gently ground on ice in an extraction buffer (0.3 M sucrose, 5 mM tetrasodium pyrophosphate, 10 mM KH_2_PO_4_, pH 7.5, 2 mM EDTA, 1% (*w*/*v*) polyvinylpyrrolidone 40, 1% (*w*/*v*) BSA, 5 mM cysteine, and 20 mM ascorbic acid). The homogenate was centrifuged at 3000× *g* for 5 min at 4 °C, and the supernatant was centrifuged once at 18,000× *g* for 30 min at 4 °C. The pellets were resuspended in wash buffer (0.3 M sucrose, 1 mM EGTA, 10 mM MOPS-KOH, pH 7.2). The protein concentration was determined using the Bradford method [73]. 

### 4.4. SDS-PAGE 

SDS-PAGE (sodium dodecyl sulfate-polyacrylamide gel electrophoresis) was performed on a Mini-Protean system (Bio-Rad, Hercules, CA, USA) as described [74] with 12% Tris-HCl gels. The gels were stained with coomassie blue [75].

### 4.5. BN-PAGE

The mitochondrial proteins were solubilized with β-DM and then separated by blue native polyacrylamide gel electrophoresis (BN-PAGE) [76]. BN-PAGE using cathode buffer blue (with 0.02% coomassie Blue G-250 added) was performed at 4 °C in a vertical apparatus. Separation gels consisted of linear gradients of 3% to 12% or 4% to 16% polyacrylamide (Invitrogen, Carlsbad, CA, USA). 

### 4.6. In-Gel Staining

The in-gel complex activity assay was performed as reported [64]. Complex I (NADH dehydrogenase) activity: 100 mM Tris-HCl, pH 7.5, 768 mM glycine, 0.1 mM NADH, 0.04% nitrotetrazolium blue (NTB) (*w*/*v*). Complex II (succinate dehydrogenase) activity: 100 mM Tris-HCl, pH 7.5, 100 mM glycine, 10 mM succinate, 0.1% NTB (*w*/*v*). Complex IV (cytochrome c oxidase) activity (for 10 mL): 5 mg 3,3′-Diaminobenzidine tetrahydrochloride (DAB) in 9 mL phosphate buffer (0.05 M, pH 7.4), 1 mL 20 μg/mL catalase, 10 mg cytochrome c, 750 mg sucrose. ATPase activity: 35 mM Tris-HCl, pH 8.0, 270 mM glycine, 14 mM MgSO_4_, 0.2% Pb(NO_3_)_2_, 8 mM ATP.

### 4.7. Western Blotting

Protein abundance was detected by Western blotting assay. BN-PAGE gels were first treated with denaturation buffer (1% SDS, 50 mM Tris-HCl, 0.05% β-mercaptoethanol) for 30 min. For Western blotting analysis, mitochondrial proteins were transferred onto PVDF membranes (0.45 mm; Millipore, Burlington, MA, USA). The PVDF membranes were incubated with various primary antibodies against wheat Nad9, maize ATP α, Arabidopsis COX2, yeast cyt *c*1, and pigeon cyt c as described previously [77,78]. Antibodies against maize A5, V1, COX3, ATPβ and ATPa were prepared in our laboratory. The rest of the primary antibodies were purchased from Agrisera company (Agrisera AB, Vännäs, Sweden). Most of these antibodies are reactive to Arabidopsis, except chlamydomonas CA2 and maize GLDH. Signal detection was carried out by ECL reagents (Thermo Fisher Scientific, Waltham, MA, USA) after incubation with the horseradish peroxidase (HRP)-conjugated secondary antibody.

### 4.8. Yeast Two-Hybrid Assay

The open reading frame (ORF) sequences of *PHB3*, *ATPa*, *ATPc*, *ATPd,* and *OSCP* were added to the expression vector pGADT7 (AD, prey). The sequences of *PHB3*, *ATPα*, *ATPβ*, *ATPγ*, *ATPδ,* and *ATPε* were cloned into the expression vector pGBKT7 (BD, bait). The resulting bait plasmids were cotransformed with prey plasmids into the yeast strains Y2H Gold (containing HIS3, ADE2, and lacZ as reporters), following the lithium acetate (LiAc)-mediated method. Transformants were grown on synthetically defined DDO medium minus Leu and Trp. The strains were screened by QDO media (lacking Leu, Trp, His, and Ade) and QDO/X-α-Gal plates with X-α-Gal. Cells transformed with the clones of p53 (pGBKT7-p53)/T-antigen (pGADT7-T) and parental empty pGBKT7/pGADT7 were used as positive and negative controls, respectively. The primers used are listed in Supplementary Table S1.

### 4.9. LCI Assay

The coding sequences of *PHB3* and genes encoding ATP synthase subunits (*ATPc*, *ATPβ*, and *ATPδ*) were cloned into vectors JW771 (nLUC) and JW772 (cLUC), respectively. The nLUC- and cLUC-related constructs were transformed into the *Agrobacterium tumefaciens* strain EHA105. Then, we mixed the agrobacterium suspensions containing the nLUC fusion and cLUC fusion in a 1:1 ratio. Both the nLUC- and cLUC-fused proteins were co-infiltrated into *N. benthamiana* leaves. After infiltration for 48 h, the leaves were soaked with 1 mM Luciferin for 10 min before imaging. The primers used are listed in Supplementary Table S1.

## 5. Conclusions

In this study, we uncovered the new roles of PHB3 in Arabidopsis mitochondria. PHB3 could interact with subunits β and δ of F1-ATPase and subunit c of Fo-ATPase. In-gel activity staining assay showed that the loss of function of PHB3 reduced the activities of ATP synthase and F1-ATPase. In the *phb3* mutant, the abundance of Fo-ATPase subunit a in ATP synthase monomer was decreased, while Fo-ATPase was accumulated. Meanwhile, when PHB3 was absent, the abundance of F1-ATPase subunits α and β was not decreased in ATP synthase, and both of them were significantly increased in F1-ATPase. These results implied that the loss of PHB3 causes the subunit a of Fo-ATPase cannot be further assembled into the intact ATP synthase. Overall, the above results of this study demonstrated that PHB3 was required for the assembly and activity of mitochondrial ATP synthase in Arabidopsis.

## Data Availability

Not applicable.

## Supplementary Materials

The following supporting information can be downloaded at: https://www.mdpi.com/article/10.3390/ijms24108787/s1.

## Abbreviations

OXPHOS, oxidative phosphorylation; CI, complex I; CII, complex II; CIII, complex III; CIV, complex IV; CV, complex V; PHB, prohibitin; β-DM, n-Dodecyl β-D-maltoside; SDS-PAGE, sodium dodecyl sulfate-polyacrylamide gel electrophoresis; BN-PAGE, blue native polyacrylamide gel electrophoresis; CBB, coomassie brilliant blue; WB, Western blotting; LCI, luciferase complementation imaging.
