# Supplementary material for: PHB3 Is Required for the Assembly and Activity of Mitochondrial ATP Synthase in Arabidopsis
Source: Int J Mol Sci. 2023 May 15;24(10):8787. doi: 10.3390/ijms24108787 (PMC10217904; doi:10.3390/ijms24108787)
Supplement: Supplementary file 1 [file ijms-24-08787-s001.zip › ijms-2370009-SI.pdf]

Table S1. List of Primers Used in This Work.

Primer names	Sequences 5’ to 3’
**Primers used for isolation of T-DNA insertion sites**	
PHB3-F	ATGGGAAGCCAACAAGCGGC
PHB3-R	TCAACGGTTCAGGGCAAAGA
LBb1.3	ATTTTGCCGATTTCGGAAC
**Primers used for Y2H vector construction**	
PHB3-F1	GGAATTCCATATGCTTTCCAATTTGGCGAAGGC
PHB3-R1	TCCCCCGGGACGGTTCAGGGCAAAGAGC
ATPa-F	ATGGCCATGGAGGCCGAATTCATGATTACTCGGCTGTTCGC
ATPa-R	CTAGTTATGCGGCCGCTGCAGCTAAATTAAAGCTAAAGCTCTTTCTTTTA
ATPc-F1	ATGGCCATGGAGGCCAGTGAATTCATGACAAAGCGTGAGTATAATTCTCA
ATPc-R1	GCAGCTCGAGCTCGATGGATTCTCAGAATACGAATAAGATCAAAAAGG
ATPd-F	ATGGCCATGGAGGCCAGTGAATTCATGAGCGGAGCCGGTAAGA
ATPd-R	GCAGCTCGAGCTCGATGGATTCTCAGTATCCCCAGTTGTCATTACG
OSCP-F	ATGGCCATGGAGGCCAGTGAATTCATGGCTAATCGTTTCAGATCAGG
OSCP-R	GCAGCTCGAGCTCGATGGATTCCAACAGGTTCACGGAGGAGC
ATPα-F	ATGGCCATGGAGGCCGAATTCATGATTACTCGGCTGTTCGC
ATPα-R	CTAGTTATGCGGCCGCTGCAGCTAAATTAAAGCTAAAGCTCTTTCTTTTA
ATPβ-F1	ATGGCCATGGAGGCCGAATTCATGGCGTCTCGGAGAGTCTTAT
ATPβ-R1	CTAGTTATGCGGCCGCTGCAGTTAAGCTGCTGACTCTTTAGCGA
ATPγ-F	ATGGCCATGGAGGCCGAATTCATGGCAATGGCTGTTTTCC
ATPγ-R	CTAGTTATGCGGCCGCTGCAGTTATTTAGCAGCTTCAAGAGCAGA
ATPδ-F1	ATGGCCATGGAGGCCGAATTCATGTTTAAACAAGCTTCTCGTCTC
ATPδ-R1	CTAGTTATGCGGCCGCTGCAGTTAGCCCGAGAGAGCTGC
ATPε-F	ATGGCCATGGAGGCCGAATTCATGGCATCGAATGCGGC
ATPε-R	CTAGTTATGCGGCCGCTGCAGTCAAACTTCAGGTGTGTCTGACC
**Primers used for LCI vector construction**	

PHB3-F2	CGGGGTACCATGGGAAGCCAACAAGCGGC
PHB3-R2	ACGCGTCGACACGGTTCAGGGCAAAGAGCATG
ATPc-F2	CGGGGTACCATGACAAAGCGTGAGTATAAT
ATPc-R2	ACGCGTCGACGAATACGAATAAGATCAAAAA
ATPβ-F2	CGGGGTACCATGGCGTCTCGGAGAGTCTT
ATPβ-R2	ACGCGTCGACAGCTGCTGACTCTTTAGCGA
ATPδ-F2	CGGGGTACCATGTTTAAACAAGCTTCTCG
ATPδ-R2	ACGCGTCGACCGCCCGAGAGAGCTGCGTTG
